# Tailoring crystallization phases in metallic glass nanorods via nucleus starvation

**DOI:** 10.1038/s41467-017-02153-4

**Published:** 2017-12-07

**Authors:** Sungwoo Sohn, Yujun Xie, Yeonwoong Jung, Jan Schroers, Judy J. Cha

**Affiliations:** 1 Department of Mechanical Engineering and Materials Science, Yale University, New Haven, CT 06511 USA; 2 Center for Research on Interface and Surface Phenomena, Yale University, New Haven, CT 06520 USA; 3 Energy Sciences Institute, Yale West Campus, West Haven, CT 06516 USA; 4 NanoScience Technology Center, Materials Science and Engineering, Electrical and Computer Engineering, University of Central Florida, Orlando, FL 32816 USA

## Abstract

Many physical phenomena deviate from their established frameworks when the system approaches relevant length scales governing the phenomena. In crystallization, the relevant length scales are the nucleation length set by the nucleus size and density, and the growth length set by diffusion fields. Here we observe unexpected crystallization phenomena at the nanoscale, using metallic glass (MG) nanorods and in situ transmission electron microscopy. The asymmetry between critical heating and cooling rates disappears for small MG nanorods. Strikingly, an apparent single crystalline phase with its composition similar to the glass composition is observed for very small rods, in contrast to bulk samples. We attribute this to the lack of nuclei in small MG nanorods that approach the nucleation length, thus coined the term, nucleus starvation. By controlling the MG nanorod diameter and crystallization kinetics, we can tune the number of nuclei in a nanorod, thereby tailoring the resulting crystallization phases.

## Abstract (web summary)

Crystallising a bulk metallic glass usually results in separate phases. Here, the authors use metallic glass nanorods to show that as the sample size approaches the nucleation scale lengths, the crystallization behavior is dictated by the lack of nuclei and nanorods crystallise into a single phase.

## Introduction

The discovery of metallic glass (MG) alloys with moderate cooling rates has motivated broad scientific and technological interests^1–3^. The ability to thermoplastically form MGs into nanostructures^4^ has further enabled direct investigation of crystallization mechanisms at the atomic scale^5^ and widened their applications, ranging from catalysts^6,7^ to bioimplants^8,9^. However, stable processing conditions at the nanoscale are largely unknown, greatly limiting their potential use. In particular, identifying the critical cooling rates is essential because they limit the formability of MGs^10,11^. The critical cooling rate, $R_{\mathrm{c}}^{\mathrm{C}}$, is defined as the lowest cooling rate to avoid crystallization when the liquid is cooled. In the same manner, the critical heating rate, $R_{\mathrm{c}}^{\mathrm{H}}$, is defined as the lowest heating rate to avoid crystallization when the glass is heated to liquid. As both rates are affected by nucleation density and nuclei size with an in inherent length scale, they may deviate significantly at the nanoscale from the values established for bulk-sized MGs.

We use Pt-based (Pt_57.5_Cu_14.7_Ni_5.3_P_22.5_) MG nanorods as a model system and observe significant deviations in the critical rates with varying nanorod diameters. We observe that the critical heating and cooling rates depend on the nanorod diameter in a non-monotonic fashion. In addition, the widely observed asymmetry between the critical heating and cooling rates disappears for MG nanorods with diameters below ~35 nm. With decreasing MG rod diameter, we also observe changes in phase formation: from the formation of expected stable constituent phases via solute partitioning, to the formation of an unexpected multi-grain, metastable phase whose composition is identical to that of the undercooled liquid, to the formation of an apparent single crystalline phase whose composition is again the same as that of the undercooled liquid. We attribute our observations to the lack of available nuclei in small MG rods as the nanorod diameter reaches the relevant nucleation length scale. In other words, the nanoscale confinement leads to nucleus starvation, similar to dislocation starvation observed in mechanical testing of small structures^12,13^. Due to the nanoscale size of the MG rods, the nuclei density can be further tuned by controlling the cooling rates of the undercooled liquid. Thus, our findings provide insight into the unexplored crystallization phases in the nanoscale regime, opening a possibility to discover emergent phases.

## Results

### Size-dependent critical heating and cooling rates

**Fig. 1:**
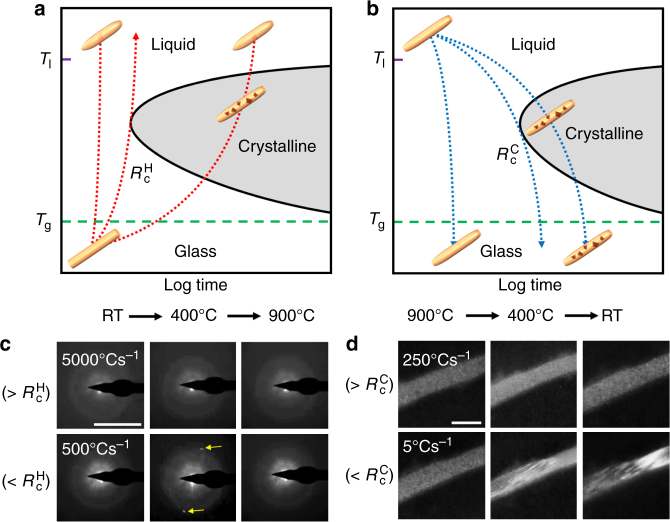
Phase transition of metallic glass nanorods upon heating and cooling using in situ TEM. **a**, **b** Schematic time–temperature–transformation (T–T–T) diagram showing the correlation between crystallization and critical rate upon heating (**a**) and cooling (**b**). The lowest heating rate that bypasses the onset of crystallization nose from the glass state to the liquid is defined as the critical heating rate ($R_{\mathrm{c}}^{\mathrm{H}}$). In the same manner, the lowest cooling rate to skip crystallization from above the liquidus temperature (*T*_l_) to the glass transition temperature (*T*_g_) is defined as the critical cooling rate ($R_{\mathrm{c}}^{\mathrm{C}}$). **c**,** d** In situ TEM characterizations of a 40 nm nanorod using SAED mode for heating (**c**) and DF mode for cooling (**d**) process to identify the critical rates. The 40 nm nanorod was repeatedly heated and cooled with various rates to identify the critical rates. **c** The snapshots show a series of SAED images obtained from room temperature (RT) to 900 °C at a constant heating rate of 5000 °Cs^−1^ (top row of **c**), Supplementary Movie 1, and 500 °Cs^−1^ (bottom row of **c**, Supplementary Movie 3), which are above and below the critical heating rate, respectively. The yellow arrows indicate the diffraction spots that reflect crystallization, which is only observed when the nanorod was heated below the critical rate. Scale bar is 5 nm^−1^. **d** The snapshots show a series of DF images taken from 900 °C to RT at a constant cooling rate of 250 °Cs^−1^ (top row of **d**, Supplementary Movie 2) and 5 °Cs^−1^ (bottom row of **d**), Supplementary Movie 4, which are above and below the critical cooling rate, respectively. The crystallization is reflected in contrast changes in the DF images, which appear only at a slow cooling rate. Scale bar is 40 nm

MG nanorods with a composition of Pt_57.5_Cu_14.7_Ni_5.3_P_22.5_ were prepared by thermoplastic forming based nanomolding^4^ and stochastic dewetting process (Supplementary Fig. 1)^5^. The crystallization kinetics of the nanorods was systematically studied by heating/cooling them inside a TEM in situ with varying heating/cooling rates (Supplementary Fig. 2). Figure 1a, b illustrates the time–temperature transformation diagrams, which show the transition between glass, crystalline, and liquid phase as a function of time and temperature. When the heating or cooling rates are lower than the critical rates, MG nanorods undergo crystallization. Above the critical rates, they remain amorphous. To visualize crystallization, we acquired in situ TEM movies of individual MG nanorods during heating or cooling. The movies were taken either in selected area electron diffraction (SAED) or dark field (DF) TEM imaging mode (Supplementary Movies 1–4). Figure 1c, d shows heating and cooling experiments of a 40 nm nanorod. When the heating and cooling rates are above the critical rates, no clear crystalline phases were detected during heating and cooling, supported by no changes in SAED patterns or in DF images (Supplementary Movies 1, 2). In contrast, when the nanorod was heated and cooled below the critical rates, a crystalline phase was observed, which is reflected as diffraction spots in the SAED pattern or a bright contrast phase in the DF image (Supplementary Movies 3, 4).

**Fig. 2:**
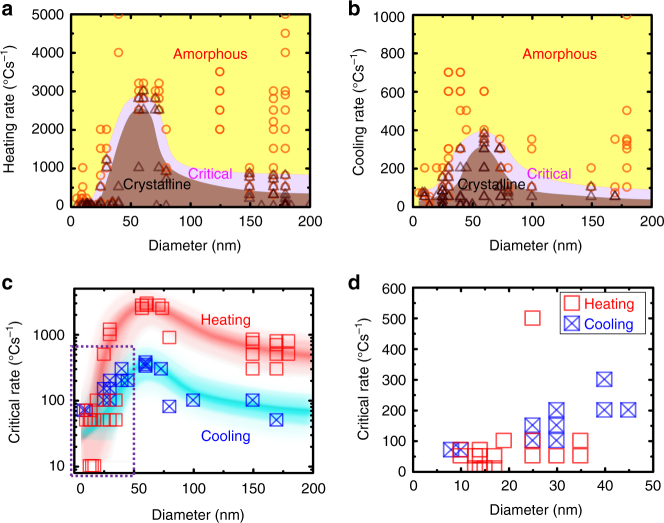
Summary of in situ TEM heating and cooling experiments for all considered nanorods. **a** Diameter-dependent critical heating rates of MG nanorods. **b**Diameter- dependent critical cooling rates of MG nanorods. Summary of the in situ TEM critical heating (**a**) and cooling (**b**) rate experiments as a function of the rod diameter. Each rod was repeatedly heated or cooled with varying rates to identify the critical rates. Orange circles indicate no observation of crystalline phases while the brown triangles indicate occurrence of crystalline phases during heating (**a**) or cooling (**b**). The regions shaded in yellow indicate that no clear crystalline phases were detected during the heating (**a**) and cooling (**b**) cycles. The regions shaded in brown indicate that crystalline phases were observed in all heating (**a**) and cooling (**b**) cycles. The critical heating (**a**) and cooling (**b**) ranges are marked in purple. **c**, **d** Critical heating and cooling range extracted from **a** and **b**. **c** The red and blue curves represent the fitted critical heating and cooling rates, respectively. The critical heating and cooling curves are fitted by the local regression model using R software. The shaded areas in red and blue indicate the scatter in the measurements of the critical rates, which reflects the stochastic nature of crystallization. **d** Magnified graph of the purple dotted rectangle in **c**, showing that the asymmetry in the critical rates disappears for MG rods smaller than ~40 nm in diameter

To determine the critical rates, the same nanorod was repeatedly heated or cooled at various ramping rates. For each rate, we determine whether or not crystallization occurs. To eliminate possible thermal history that could affect the experiments, the nanorod was always heated to 900 °C for <5 s before each run (Supplementary Fig. 2). Summary of the experiments for all considered nanorods is shown in Fig. 2a and b for heating and cooling, respectively. Experiments denoted with circles and triangles indicate the absence and presence of crystalline phases during heating or cooling. Due to the stochastic nature of nucleation^14–16^, the critical heating and cooling rates are usually expressed as a range instead of a single value^17^. Thus, the critical ranges are identified as the regions where the circle and triangle data points overlap. At the critical range, we performed multiple heating and cooling experiments to ensure that the overlapped region accurately marks the boundary between the circle and triangle data points. Surprisingly, the critical ranges are not constant, and they deviate significantly from the bulk value when the diameter is below 100 nm. Both the critical heating and the cooling range increase with decreasing diameter, exhibit a maximum at ~60 nm, and then rapidly decrease with decreasing size. This non-monotonic behavior is in agreement with our previous study that showed nanoscale confinement effects on crystallization temperature^5,18^. Namely, for rods below ~60 nm, the low probability of nucleation and decrease in the growth rate dominate over heterogeneous nucleation to reduce the crystallization kinetics^5^. The increase in the critical rates for rods larger than ~60 nm in diameter is attributed to the enhanced heterogeneous nucleation due to the large surface to volume ratios of nanorods. Extrinsic factors that could potentially affect crystallization during our in situ TEM experiments, such as chemical composition change, e-beam irradiation, surface oxidation, carbon build-up, nanorod curvature effects, and thermal conductivity in reduced dimensions, have been ruled out (Supplementary Note 1)^5^.

We observe a significant asymmetry between the critical heating and cooling range. The critical heating range is about one to two orders of magnitude larger than the cooling range, in agreement with the previous crystallization kinetics study for a bulk MG^19–22^. This asymmetry is shown clearly in Fig. 2c, which shows only the critical heating and cooling range curves. According to classical nucleation and growth theory, this asymmetry in the critical rates originates from dissimilar temperature dependence of nucleation and growth rates, where the maximum nucleation rate occurs at a lower temperature than the maximum growth rate (Supplementary Note 2; Supplementary Fig. 3)^19,23^. Thus, crystallization upon heating a glass would contain more nuclei than crystallization upon cooling a stable liquid. The large number of nuclei formed during heating the glass subsequently grow upon heating, leading to faster crystallization kinetics than crystallization from the undercooled liquid that does not contain as many nuclei^19,24^. Surprisingly we found that this asymmetry disappears for rods smaller than ~35 nm in diameter (Fig. 2d). Such disappearance in asymmetry has never been reported before. Because the origin of the asymmetry stems from the disparity in the number of nuclei, its disappearance suggests the lack of nuclei in small rods. In addition to the nanoscale size that limits the number of nuclei, the decreased nucleation rate and the more rapid cooling rates for smaller rods also lead to a smaller number of nuclei in these small nanorods, contributing to the merge of the critical heating and cooling rates. We also note that increase in viscosity^18^ and decrease in growth rates^5^ have been observed in these nanorods. The lower atomic mobility at the nanoscale could also contribute to the decrease in the asymmetry between the heating and cooling rates.

### Crystalline phases by nanoscale confinement and kinetics

**Fig. 3:**
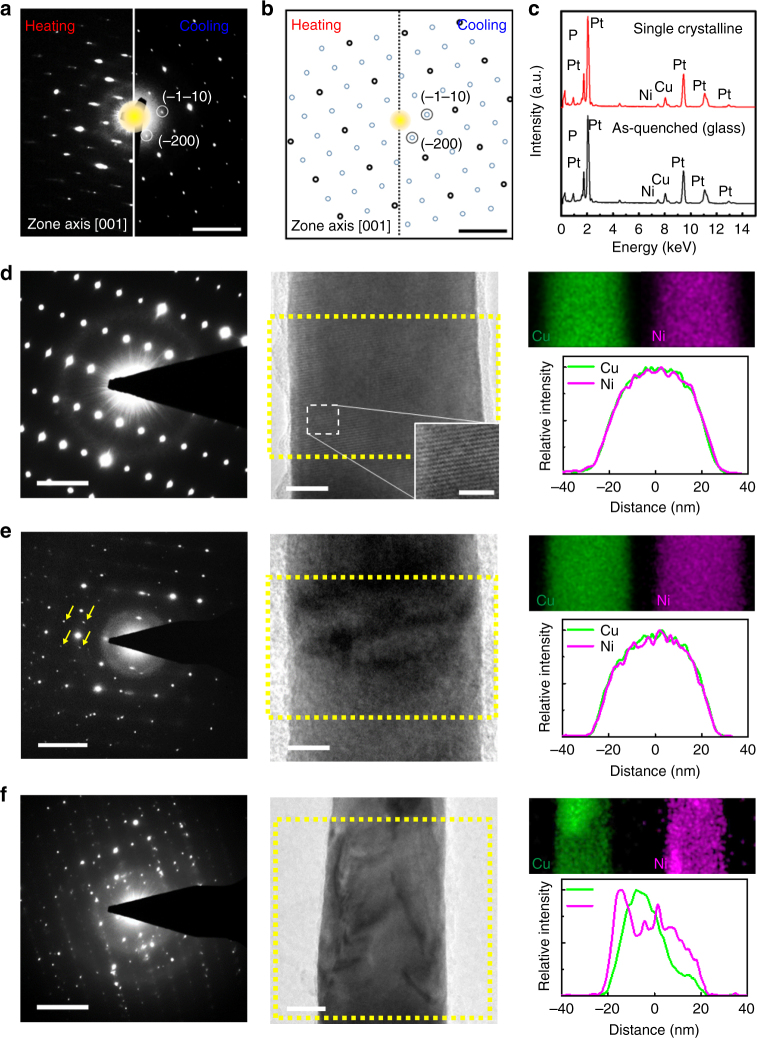
Multiple crystallization phases as a function of the cooling rate and the metallic glass nanorod diameter. **a**, **b** Characterization of the apparent single crystalline phase upon heating and cooling of the MG nanorods. **a** SAED patterns obtained during heating (100 °Cs^−1^) (left half of **a)** and cooling (50 °Cs^−1^) (right half of **a**) of a 40 nm nanorod. Both patterns, identical to each other, show a [001] zone axis of a monoclinic C2/c-like structure. Detailed crystal structure discussion in Supplementary Note 3. **b** Schematic representation of the SAED pattern, indicating the same crystalline product during the heating and cooling. **c** EDX spectra from the apparent single crystalline phase and the glass phase of the nanorod. The spectra show negligible differences, suggesting identical chemical composition for the single crystalline and the glass phase. **d** An apparent single crystalline phase in a 45 nm nanorod cooled at a higher cooling rate (100 °Cs^−1^). The single crystallinity is confirmed by the SAED pattern (left panel of **d**) and lattice fringes in the TEM image (middle panel of **d**), scale bar = 10, 2 nm in inset). Its chemical composition appears identical to the glass composition with the Ni and Cu homogeneously distributed (right panel of **d**). **e** A polycrystalline grain in a 45 nm nanorod cooled at a lower cooling rate (5 °Cs^−1^). The SAED pattern shows a polycrystalline nature with extra diffraction spots pointed by yellow arrows (left panel of **e**). The bright and dark contrast change in the TEM image (middle panel of **e**, scale bar = 40 nm) suggests a relative orientation difference of the grains. Its chemical composition appears identical to the glass composition with the Ni and Cu homogeneously distributed (right panel of **e**). **f** A polycrystalline grain in a 80 nm nanorod cooled at a lower cooling rate (5 °Cs^−1^). The SAED pattern shows crystalline grains with various orientations (left panel of **f)**. Scale bar = 25 nm in the middle panel of **f**. The chemical compositions of the polycrystalline grains are different from the glass composition, as evident by the Cu-rich and Ni-rich regions (right panel of **f**). All scale bars in SAEDs are 4 nm^−1^

The reduced number of nuclei in small rods suggests that it may be possible to crystallize a small MG rod into a single crystalline phase if the nuclei density can be controlled. Indeed, during the critical cooling rate experiments, we observe an unexpected single crystallinity in a MG rod. Figure 3a shows SAED patterns obtained from a 40 nm nanorod during heating (left half) and cooling (right half) experiments with ramping rates lower than the critical rates. The diffraction patterns look identical between the heating and cooling, and suggest single crystallinity. Considering the symmetry of the diffraction patterns, we tentatively assign the crystalline phase to adapt a C2/c-like structure of a platinum phosphide (P_2_Pt_5_) phase (crystal structure discussion in Supplementary Note 3). Due to the small size of the nanorod, obtaining diffraction patterns along different crystallographic orientations was challenging, making it difficult to definitively confirm the single crystalline phase. Thus we will denote it as an apparent single crystalline phase. Surprisingly, the apparent single crystalline phase appears to have the same chemical composition to the glass composition (Fig. 3c), examined by energy dispersive X-ray spectroscopy (EDX). This suggests that the apparent single crystalline phase is a metastable phase.

To investigate this further, we cooled nanorods of different diameters at varying cooling rates and examined their resulting crystalline phases. We observe three distinct crystallization phases: the apparent single crystalline phase as mentioned earlier, a polycrystalline phase with the chemical composition identical to that of the undercooled liquid, and a polycrystalline phase with multiple constituent phases via solute partitioning. Nanorods with similar diameters can be crystallized into different crystallization phases when the cooling rate is different. At a higher cooling rate (100 °Cs^−1^), a nanorod (~45 nm diameter) crystallizes into the apparent single crystalline phase, as suggested by the ordered SAED pattern and lattice fringes (Fig. 3d). At a lower cooling rate (5 °Cs^−1^), another nanorod with a similar diameter (~45 nm) crystallizes into a polycrystalline phase (Fig. 3e). This is supported by extra spots in SAED (marked by arrows in Fig. 3e, left panel) that indicate a presence of another crystalline phase, and a visible contrast change in the middle of the bright field (BF) image (Fig. 3e, middle panel), which implies a relative orientation difference of the grains. Notably for both cases (Fig. 3d, e), the composition of the crystalline phase shows no detectable differences from that of the liquid phase, and the composition is homogeneous throughout the crystalline phase, indicating no phase segregation, as observed by EDX mapping of Cu and Ni (Fig. 3d, e, right panels). A relatively larger nanorod (~80 nm) with a low cooling rate (5 °Cs^−1^) shows multiple crystalline grains with chemical heterogeneity. These multiple crystalline grains are reflected in complex ordering in the SAED pattern (Fig. 3f, left panel) and contrast variations in the BF image (Fig. 3f, middle panel). The chemical heterogeneity among different phases is captured in EDX mapping (Fig. 3f, right panel), which shows Cu-rich and Ni-rich regions, which is typically observed in bulk MGs.

## Discussion

**Fig. 4:**
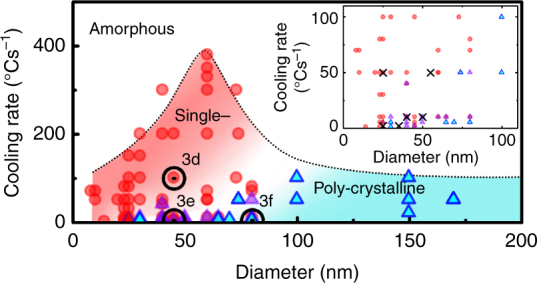
Tailoring crystallization phases of nanorods with a diameter-dependent critical cooling rate. A diagram of a diameter-dependent critical cooling rate of MG nanorods to show tunable crystallization phases. Red circles, purple triangles, and blue triangles indicate an apparent single crystalline phase, a polycrystalline phase with no detectable chemical compositional difference from the glass phase, and a polycrystalline phase with different chemical compositions, respectively. The black circles indicate crystallization phases shown in Fig. 3d–f. A dotted black line is to show the critical cooling rate, fitted by the local regression model using R software. (Inset) A magnified graph of the small diameter (<100 nm) and low cooling rate regime (<100 °Cs^−1^). Black crossed marks indicate observation of partial crystallization with solute partitioning

Figure 4 summarizes all the observed crystalline phases, i.e., solidification products. A phase diagram emerges that marks the transition between the polycrystalline phase and the apparent single crystalline phase as a function of the nanorod diameter and the cooling rate. Red circles denote all the cooling experiments that have resulted in the apparent single crystalline phase, while blue triangles denote all the cooling experiments that have resulted in a polycrystalline phase. The inset zooms into the region with smaller diameters (<100 nm) and slower cooling rates. This transition at the nanoscale, particularly the observation of the apparent single crystalline phase, has not been observed previously. Previous studies on bulk MGs report multiple (meta-) stable constituent phases during crystallization. For the studied Pt-based MGs^25^, they are P_2_Pt_5_, CuP_2_, NiP_2_, and NiPt. We find two factors that critically influence the apparent single crystallization phase: a fast cooling rate in which kinetics dominate over thermodynamic stability and the nanorod diameter that approaches the length scale of the nuclei density, i.e., the average mean distance between nuclei. Thus, we hypothesize that the formation of the apparent single crystalline phase must be kinetically driven, far away from thermodynamic equilibrium. As the cooling rate increases, the absolute time for crystallization decreases, reducing the number of nucleation events. Thus, at a higher cooling rate, only a single nucleation event may occur, which would result in a single crystalline grain, as shown in Fig. 3d. In addition, this would occur more likely in nanoscale samples whose size approaches the mean distance between critical nuclei. At a lower cooling rate, more time is given to form multiple nuclei, which would result in a polycrystalline phase (Fig. 3e or f).

EDX spectra in Fig. 3c show that the chemical composition of the apparent single crystalline phase appears the same as the glass composition even though, for this MG composition, four distinct constituent phases exist. Thus, the observed single crystalline phase must be metastable. This is possible because rules of alloy phase equilibria that apply typically to macroscopic samples can be violated at the nanoscale^26^. Thermodynamically, competition between a metastable crystalline phase and an amorphous phase may be possible in nanoscale systems due to Gibbs free energy variations from interfacial effects, such as interfacial segregation and boundary stress^26,27^. In addition, liquid–solid interfacial energy values for metastable phases are generally less than that for stable phases^28,29^, which helps the formation of a metastable phase^30^. Furthermore, fast cooling prefers kinetically trapped, metastable phases to thermodynamically stable phases by virtue of effective suppression of stable nuclei formation^31–33^. Consequently, no distinct enrichment or depletion in solute partitioning has been observed in formation of the kinetically favored, metastable phase. For larger nanorods cooled with slow cooling rates, we observe polycrystalline grains with Cu- and Ni-rich regions, as expected (Fig. 3f).

The emergence of the apparent single crystalline phase with its chemical composition identical to that of the glass marks a clear departure from the expected solidification products in this multi-component, Pt-based MG alloy. This is akin to the surprising departure of mechanical properties in small microscale to nanoscale metallic structures from the expected mechanical properties in bulk samples^12,13^. The origin of the mechanical property deviation at the micro to nanoscale was attributed to the lack of dislocations in samples, coined as dislocation starvation^12,13^. Likewise, we define the lack of nuclei that leads to the apparent single crystalline phase as the nucleus starvation. In addition, the observed increased viscosity^18^ could also contribute to the emergence of the metastable, single crystalline phase.

**Fig. 5:**
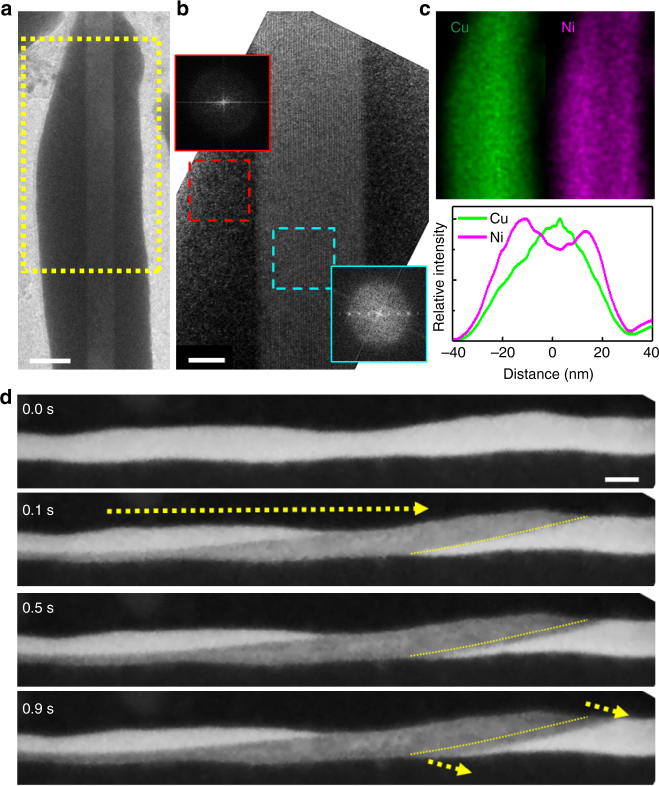
Observation of partially crystallized nanorods with solution partitioning upon cooling. **a**, **b** A 55 nm partially crystallized nanorod (**a**), obtained by a constant cooling rate of 10 °Cs^−1^. A crystalline core, of which the width and length dimensions are 20 nm and over 330 nm, is shown to be single crystalline surrounded by an amorphous shell (**b**). Scale bars are 25 and 6 nm, respectively in **a** and **b**. The Fourier transformed images (diffractograms) obtained from the core crystalline region (cyan dashed square) and the amorphous shell (red dashed square) confirm the single crystalline and the amorphous nature, respectively. **c** Cu and Ni maps obtained from the yellow dashed region in **a** are shown with their relative intensity profiles. The crystallized core is Cu-rich and simultaneously Ni-poor. **d** Snapshot DF TEM images of a 25 nm nanorod from an in situ movie (Supplementary Movie 5). The growth of the single crystalline core during a constant cooling (50 °Cs^−1^) is captured by tracing the expansion of the crystalline growth front, reflected as the change of imaging contrast (yellow dashed lines). Scale bar = 30 nm

The more common solute partitioning is still observed in small MG nanorods, although its occurrence is rarer than the apparent single crystalline phase. Black crossed marks in the inset of Fig. 4 indicate observations of the solute partitioning during crystallization. Figure 5a shows an example of a core-shell MG nanorod upon crystallization during cooling. The core is a single crystalline phase while the shell is amorphous. The single crystalline core is confirmed by high-resolution TEM imaging and Fourier transforms (Fig. 5b). The Fourier transform of the shell shows its amorphous nature. Unlike the apparent single crystalline phase that shows the same composition as the glass composition, here the observed single crystalline core is Cu-rich, as shown by the EDX map (Fig. 5c). The amorphous shell is Ni-rich as the Ni atoms are ejected out from the crystalline core during crystallization. The Ni-rich shell remains amorphous even though the glass forming ability is sensitive to the alloy composition. This is likely because the barrier to nucleate a crystalline phase is too high in the Ni-rich chemical composition. This is further supported by the slow growth rate in the radial direction, which can be associated with the effect of solution partition. The growth of the single crystalline core is directly captured in real time in the in situ TEM movie (Supplementary Movie 5). Figure 5d shows a series of snapshots from a different nanorod that demonstrates a similar behavior. The dark contrast region in the center indicates a single crystalline phase sandwiched by an amorphous phase. Surprisingly, most of the crystallization occurs within 0.1 s, with the grain length on the order of ~500 nm and width of ~20 nm.

The drastically different solidification products observed here in MG nanorods highlight the complexity of crystallization at the nanoscale^34–36^, which marks a departure from crystallization framework established for samples whose size are is compared to intrinsic length scales. The significant deviation in crystallization kinetics at the nanoscale is most pronounced by the maxima in critical heating and cooling rates as a function of the nanorod diameter. Our in situ crystallization experiments of individual MG nanorods inside a TEM provide a unique opportunity to build atomic understanding of the early stages of crystallization.

## Methods

### Preparation of metallic glass nanorods

The Pt_57.5_Cu_14.7_Ni_5.3_P_22.5_ MG nanorods were prepared by the nanomolding technique described in our previous report^4^. First, a master alloy, ~20 g, was prepared by alloying high-purity elements (>99.95) with nominal compositions in an evacuated quartz tube (to 1 Pa ≈ 7.5 mTorr) using an induction melting system. The alloy was then fluxed with dehydrated boron trioxide (B_2_O_3_, ~10 g) at 1200 °C, which is 450 °C above the liquidus temperature for Pt_57.5_Cu_14.7_Ni_5.3_P_22.5_, for 30 min to remove impurities. Subsequently, the fluxed alloy was re-melted at 1100 °C for 2 min and vitrified by water quenching. To fabricate nanorods from the bulk alloy, a commercially available anodized aluminum oxides (AAO) with pore sizes ranging from 13 to 200 nm were used as a mold (Synkera Inc.). The nanomolding fabrication was carried out in a custom-built heating cell equipped with the Instron 5569 machine. A piece of MG plate, ~2 mm in height, was positioned on the mold and equilibrated at 260 °C for 30 s. Nanorods were molded by pressing the MG plate into the selected AAO mold under a load ramping from 0 to 100 kN for 2 min at 260 °C. The formed nanorods still attached to the MG plate were collected by dissolving the AAO mold using a 20 wt.% potassium hydroxide (KOH) solution heated at 80 °C for ~10 h, and the sample was repeatedly rinsed with distilled water and isopropanol (IPA) for more than four times. The nanorods were detached from the MG plate by sonication. The smallest available pore size for AAO molds was 13 nm.

### In situ TEM experimental details

In situ TEM experiments were performed with FEI Tecnai Osiris 200 kV TEM at Yale. Thermoplastically formed nanorods dispersed in IPA were transferred to in situ thermal chips via drop casting. A commercially available in situ TEM system was used for heating and cooling (Aduro 300DT System by Protochips Inc). The TEM thermal grids consist of a holey silicon nitride (Si_3_N_4_) substrate with an overlaid thin amorphous carbon film, and metal electrodes, which heat the membrane via Joule-heating. Nanorods sitting on the film were monitored in real time, while the temperature was controlled with pre-calibrated parameters provided by the manufacturer. In situ TEM movies were video-recorded (Snagit Software) either in diffraction mode or in TEM BF/DF imaging planes. To eliminate any effects thermoplastic forming would have on crystallization experiments of the MG nanorods and to start crystallization experiments from the same melt state, we first heated nanorods up to 900 °C, ~300 °C higher than the liquidus temperature of nanorods, for a short time. For the critical heating rate experiments, we rapidly quenched the nanorods from 900 °C down to room temperature with a maximum cooling rate of ~10^6^ °Cs^−1^, and then heated the nanorod up to 900 °C at the selected ramp rate (Supplementary Fig. 2a). For the critical cooling rate experiments, we cooled the nanorods from the initial 900 °C at a controlled cooling ramp rate and monitored the nanorod in real time (Supplementary Fig. 2b). The temperature of the nanorods was assumed to be the same as the temperature of the in situ TEM thermal grids, whose temperature was read out by the power controlling system.

### Data availability

All raw data presented in this work are available from the corresponding authors upon request.

## Electronic supplementary material

Supplementary Information
