# Supplementary material for: Tailoring crystallization phases in metallic glass nanorods via nucleus starvation
Source: Nat Commun. 2017 Dec 7;8:1980. doi: 10.1038/s41467-017-02153-4 (PMC5719396; doi:10.1038/s41467-017-02153-4)
Supplement: Supplementary file 1 — Supplementary Information [file 41467_2017_2153_MOESM1_ESM.pdf]

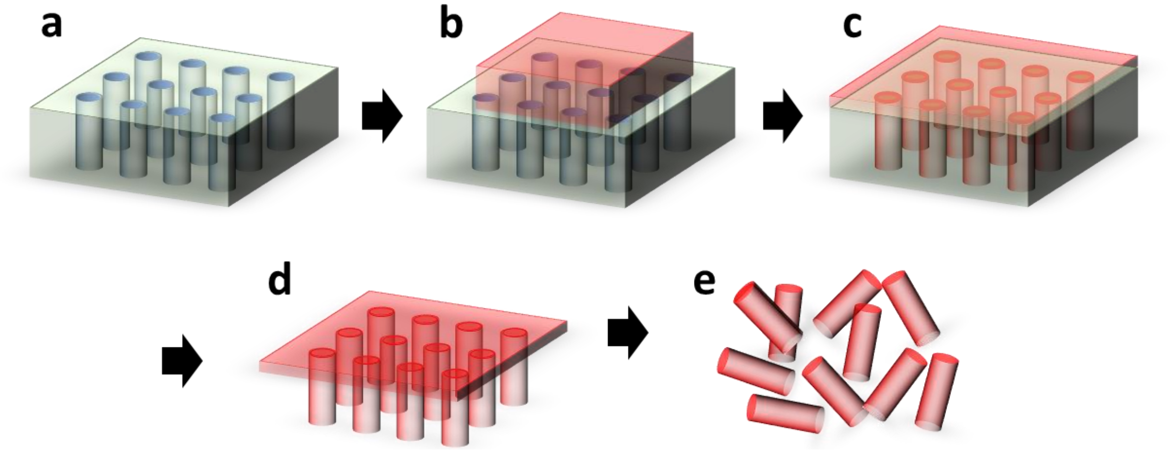

Supplementary Figure 1. Preparation of MG nanorods from a bulk alloy. **a.** A commercially available anodized aluminum oxides (AAO) mold with cavities. **b.** Place a bulk alloy on the mold and heat at 260°C for 30 sec. **c.** Thermoplastic forming of the bulk alloy into the AAO mold under a load ramping from 0 to 100 kN in 2 mins at 260°C. **d.** Releasing nanorods attached on a MG plate by dissolving the AAO mold using a 20 wt.% potassium hydroxide (KOH) solution. **e.** Detaching nanorods from the MG plate by sonication.

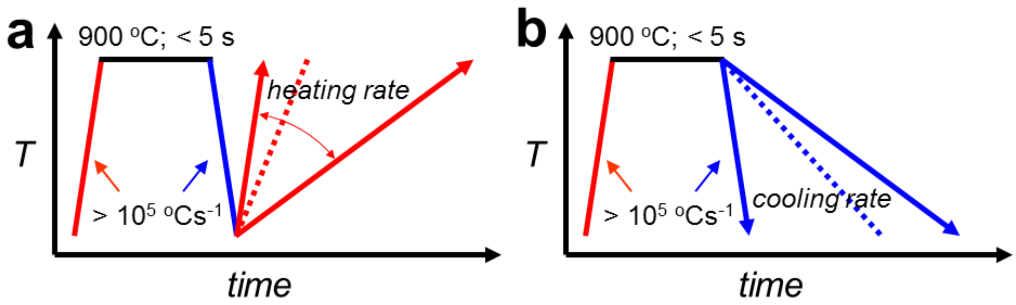

Supplementary Figure 2. Temperature-time steps used for size-dependent critical rate experiments. **a.** The MG nanorods were first rapidly heated to 900°C for < 5 sec, then quenched to room temperature to remove any previous thermal history. 900°C is well above the melting temperature of $Pt_{57.5}Cu_{14.7}Ni_{5.3}P_{22.5}$ MG (for bulk sample, T_m = ~ 500°C). After the initial heat-quench step, the rods were heated at a specific heating rate during which crystallization was monitored. Per rod, we repeated the heating experiment many times with varying heating rates to determine the critical heating range. **b.** The temperature-time steps for critical cooling experiments. In this case, after the rapid anneal to 900°C, the molten rods were cooled at a specific cooling rate during which crystallization was monitored. Per rod, we repeated the cooling experiment many times with varying cooling rates to determine the critical cooling rate.

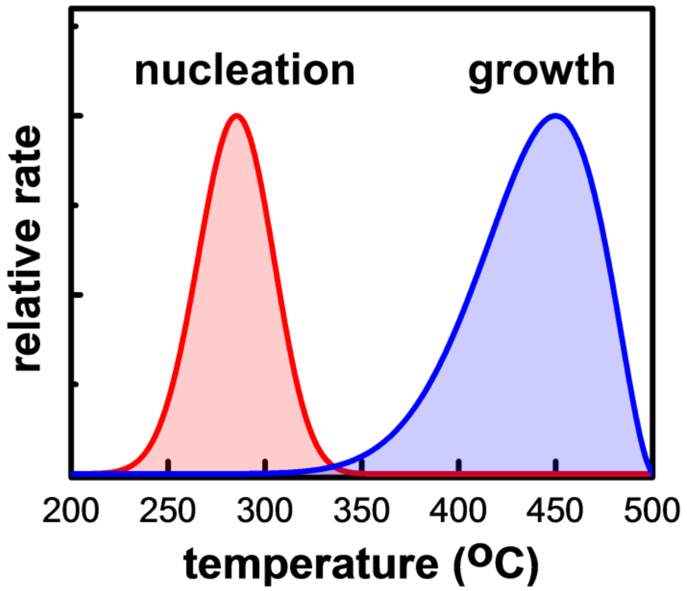

Supplementary Figure 3. Calculation of the temperature ranges for the maxima of nucleation and growth rates based on classical theory of a Pt-based BMG ($Pt_{57.3}Cu_{14.6}Ni_{5.3}P_{22.8}$). The maximum nucleation rate occurs at a lower temperature than the maximum growth rate. This mismatch in the temperature ranges between nucleation and growth has been used to explain the widely observed crystallization asymmetry from the glass or the liquid phase for bulk MGs. All parameters are collected from the previous study using a differential scanning calorimeter (ref 8).

Supplementary Note 1: Possible extrinsic experimental factors that can affect crystallization kinetics

We consider *extrinsic* experimental factors that can potentially affect crystallization kinetics when observing nanorod samples using TEM. These factors include composition change during experiments, electron beam (e-beam) irradiation, effects of a surface oxidation layer, carbon build-up, the curvature of the nanorod effect, reduced thermal conductivity for nanoscale samples, and potentially poor thermal contact between nanorods and the thermal TEM grid. We discuss how these factors would have affected crystallizations, and rule out the possibility that they have any significant effects on the crystallization kinetics of the rods we studied based on our observations.

Composition effects. To the best of our knowledge, there are no measurement techniques, including *in situ* TEM, which can measure chemical compositions of nanoscale samples in a non-destructive, *in situ*, spatially-resolved and quantitatively accurate fashion. Energy dispersive X-ray spectroscopy was used to measure the composition of the rods before and after experiments, which show negligible differences between spectra. Thus, we can only infer how chemical compositions would have affected the crystallization kinetics. If chemical compositions changed as we repeated *in situ* TEM experiments, the crystallization kinetics should change with repeated heating/cooling cycles. If chemical compositions were stable, we should obtain cycle number independent results. To verify the critical heating and cooling range, we performed multiple heating and cooling cycles, at least 5 times per each condition per nanorod, to ensure this range accurately marks the boundary. At the critical range, we sometimes observe crystallization and other times not. Based on the collected data (~ 250 experiments in total), we could not find any correlation, in the statistical sense, between the

number of cycles and crystallization kinetics. This finding, that crystallization kinetics does not deviate with repeated experiments, agrees with our previous cyclic heating experiments[1] with a ramp rate of 0.67°Cs^{-1}, which was performed over 50 times (Supplementary Notes in [ref 1]), showing a reliable crystallization onset temperature (T_c) with a standard deviation of 1.5°C. This observation of reliable crystallization temperature as well as reliable critical heating and cooling ranges regardless of how many experiments were repeated points out that compositional changes, if present, are negligible. Also, the stable crystallization kinetics upon repeated experiments suggest negligible e-beam effects, carbon build-up, and surface oxidation effects.

E-beam irradiation effects. We had taken into account the e-beam effect in our previous study[1]. Briefly, to test potential electron beam effects on crystallization kinetics, after 50 heating cycles which took ~ 6 hours, we measured another nanorod of the similar diameter, which was located on a different part of the thermal chip, thus was not illuminated with the e-beam. The T_c was only 1.7°C away from the average T_c of the nanorod that was subject to the heat cycles. This result indicates that the relative temperatures are not affected by the e-beam illumination. We note that the absolute temperature value or growth rate may be subject to some shift with the beam-off conditions. However, our main findings of the non-monotonic trend of the critical heating and cooling rates, and vanishing asymmetry in crystallization from the liquid and the glass phase are unaffected by the e-beam irradiation because they are mainly based on relative temperature differences. Thus, we conclude that any e-beam effects cannot explain our findings.

Surface oxidation or carbon build-up effects. One of the major obstacles to form a glass is heterogeneous nucleation[2,3]. Especially, in noble metal based MG systems, e.g. Pd-based MGs[4] and Pt-based MGs[5], reducing the heterogeneous surface nucleation sites is central to obtaining a glass state. Thus, we consider possible oxidation effects that could influence our *in situ* TEM

experiments. First, the possibility that the nanorods will be oxidized overtime inside a TEM is minimum due to repeated heat cycling up to 900°C and the high vacuum of the TEM column. If surface oxidation were to occur over time during *in situ* experiments, again we would observe that the critical heating and cooling rates would drift gradually with repeated experiments. We do not observe such trend. In addition, the effect of a surface oxide layer potentially accelerating crystallization kinetics would be greater for smaller rods due to the larger surface-to-volume ratio. However, we observe much decelerated crystallization kinetics for small nanorods compared to the large nanorods. Thus, we conclude that surface oxidation or carbon build-up is minimal.

Curvature effects. Now, we discuss a potential curvature effect, the so-called Gibbs-Thomson effect. In our previous report[1], we discuss the presence of the Gibbs-Thomson effect in detail (Supplementary Notes in [ref 1]). We measured the melting (T_m) and liquidus (T_l) temperature of the nanorods as a function of the nanorod diameter ranging from ~ 4 nm to ~ 57 nm. The results show monotonous decrease of T_m and T_l. The T_m of the smallest sample (~ 4 nm) is reduced by as much as ~ 130°C from the bulk value. From the data, we conclude that the magnitude of the Gibbs-Thomson effect alone cannot explain the observed non-monotonic behavior in critical heating and cooling rates and the vanishing of the asymmetry in crystallization from the melt or the glass state as a function of the nanorod diameter.

Thermal conductivity of nanoscale samples. If the thermal conductivity decreases significantly, or the thermal contact becomes poor/unreliable for smaller nanorods, we hypothesize that the T_m and T_l should also increase due to poor heat transfer between the heater and the sample, or at least should not follow the prediction of the Gibbs-Thomson effect. Our previous results[1] show

that both the T_m and T_l decrease with decreasing diameter, as expected from the Gibbs-Thomson effect. This indicates that the decrease in thermal conductivity is insignificant.

Supplementary Note 2: The established classical crystallization theory to explain crystallization asymmetry

When a liquid is undercooled below its melting temperature, a thermodynamic driving force, i.e. the Gibbs free energy difference between the liquid phase and the crystal phase, dictates crystallization, which can be described by the classical theory[4,6,7]. According to the classical theory, crystallization requires two steps, the formation of nuclei and subsequent crystal growth of the nuclei. Based on this concept, Johnson-Mehl-Avrami (JMA) framework developed in 1930's has been used to describe crystallization phenomena in glassy materials. According to the JMA kinetic theory, the crystallization fraction in glasses undergoing crystallization is expressed as[6];

$$X = 1 - exp\left[-\frac{\pi}{3}u^3 I t^4\right] \tag{1}$$

where X is the transformed fraction, t is time for transformation, I is nucleation frequency per unit volume (nucleation rate) and u is growth rate of the phase-transforming interface per unit area of the interface. In this classical framework, the nucleation rate (I) is a product of a concentration of nucleation sites, an effective diffusivity and the thermodynamic Boltzmann factor of the energy barrier[6]. Here, the effective diffusivity strongly depends on temperature, which decreases with decreasing temperature. The energy barrier is required to generate a solid-liquid interface. Due to the balance between the diffusivity and the energy barrier, the maximum nucleation rate generally occurs at a lower temperature (red curve in Supplementary Figure 3)

than the maximum growth rate which only depends on the diffusivity and the driving force (blue curve in Supplementary Figure 3). This mismatch in the temperature range for the maximum nucleation rate and growth rate has been used to explain the widely observed asymmetry in the critical heating and cooling rates of metallic glasses. For critical heating rates, as the glass is heated to crystallize, the maximum nucleation rate is accessed first to create a large number of nuclei, which subsequently grow. For critical cooling rates, as the liquid is cooled to crystallize, the maximum nucleation rate is not accessed before the maximum growth rate, thus crystallization occurs much more slowly. Thus, the critical heating rate is usually higher than the critical cooling rate. Based on the previous study on the $Pt_{57.3}Cu_{14.6}Ni_{5.3}P_{22.8}$ alloy[8], we have calculated the temperature ranges for the maxima of nucleation and growth rates (Supplementary Figure 3).

Supplementary Note 3: Possible crystal structure and crystallization mechanism of $Pt_{57.5}Cu_{14.7}Ni_{5.3}P_{22.5}$ MG nanorods

Based on the previous report[1], the two competing crystalline products for the $Pt_{57.5}Cu_{14.7}Ni_{5.3}P_{22.5}$ MG alloy are platinum phosphide (P_2Pt_5) with monoclinic C2/c structure and copper phosphide (CuP_2) with P21/c structure. We use selected area electron diffraction (SAED) patterns to examine the crystalline structure of our MG nanorods. Figure 3a in the main text shows SAED patterns obtained from a crystallized 40 nm nanorod after heating (heated up from room temperature (RT) to 400^oC with a ramp rate of 100$^oCs^{-1}$ and quenched for structural analysis) and cooling (quenched down from 900^oC to RT with a ramp rate of 50$^oCs^{-1}$) experiments. Figure 3b shows the corresponding schematics. Both SAED patterns suggest a formation of the C2/c structure in [001] zone axis. To distinguish between the two crystalline structures, C2/c and

P21/c, we examine the symmetry of the SAED patterns. The symmetry of C2/c is 63.13 degrees between (-200) and (-1-10) spots, whereas that of P21/c is 90 degrees. In our case, the SAED patterns show 65 degree symmetry, which is much closer to C2/c structure. We note that analyzing the lattice constant of the crystallized MG rod is not so helpful in determining the crystal structure because of similar lattice constants of the two phases (for example, d_{200} of C2/c and d_{100} of P21/c are 5.32 Å and 5.35 Å, respectively). We also note that the diffraction patterns show single crystallinity with less than ~ 10 % difference in the lattice constants compared to the known standard C2/c structure from the diffraction data library (PDF#23-0465).

Supplementary References

1. Sohn, S. *et al.* Nanoscale size effects in crystallization of metallic glass nanorods. *Nat. Commun.* **6**, 8157 (2015).
2. Kui, H. W., Greer, A. L. & Turnbull, D. Formation of bulk metallic glass by fluxing. *Appl. Phys. Lett.* **45**, 615-616 (1984).
3. Onorato, P. I. K. & Uhlmann, D. R. Nucleating heterogeneities and glass formation. *J. Non-Cryst. Solids* **22**, 367-378 (1976).
4. Inoue, A., Nishiyama, N. & Kimura, H. Preparation and thermal stability of bulk amorphous $Pd_{40}Cu_{30}Ni_{10}P_{20}$ alloy cylinder of 72 mm in diameter. *Mater. Trans. JIM* **38**, 179-183 (1997).
5. Schroers, J. & Johnson, W. L. Highly processable bulk metallic glass-forming alloys in the Pt-Co-Ni-Cu-P system. *Appl. Phys. Lett.* **84**, 3666-3668 (2004).
6. Uhlmann, D. R. A kinetic treatment of glass formation. *J. Non-Cryst. Solids* **7**, 337-348 (1972).
7. Schroers, J., Masuhr, A., Johnson, W. L. & Busch, R. Pronounced asymmetry in the crystallization behavior during constant heating and cooling of a bulk metallic glass-forming liquid. *Phys. Rev. B* **60**, 11855-11858 (1999).
8. Legg, B. A., Schroers, J. & Busch, R. Thermodynamics, kinetics, and crystallization of $Pt_{57.3}Cu_{14.6}Ni_{5.3}P_{22.8}$ bulk metallic glass. *Acta. Mater.* **55**, 1109-1116 (2007).
